# Impact of a Mobile Phone App to Increase Vegetable Consumption and Variety in Adults: Large-Scale Community Cohort Study

**DOI:** 10.2196/14726

**Published:** 2020-04-17

**Authors:** Gilly A Hendrie, M Sazzad Hussain, Emily Brindal, Genevieve James-Martin, Gemma Williams, Anna Crook

**Affiliations:** 1 Nutrition and Health Program CSIRO Health & Biosecurity Adelaide Australia; 2 The Australian eHealth Research Center CSIRO Health & Biosecurity Sydney Australia; 3 SP Health Co. Pty Ltd Sydney Australia

**Keywords:** mHealth, vegetables, healthy diet, intervention study

## Abstract

**Background:**

Large-scale initiatives to improve diet quality through increased vegetable consumption have had small to moderate success. Digital technologies have features that are appealing for health-related behavior change interventions.

**Objective:**

This study aimed to describe the implementation and evaluation of a mobile phone app called VegEze, which aims to increase vegetable intake among Australian adults.

**Methods:**

To capture the impact of this app in a real-world setting, the Reach, Effectiveness, Adoption, Implementation, and Maintenance framework was utilized. An uncontrolled, quantitative cohort study was conducted, with evaluations after 21 and 90 days. The app was available in the Apple App Store and was accompanied by television, radio, and social media promotion. Evaluation surveys were embedded into the app using ResearchKit. The primary outcomes were vegetable intake (servings per day) and vegetable variety (types per day). Psychological variables (attitudes, intentions, self-efficacy, and action planning) and app usage were also assessed. Descriptive statistics and multiple linear regression were used to describe the impact of the app on vegetable intake and to determine the characteristics associated with the increased intake.

**Results:**

Data were available from 5062 participants who completed the baseline survey; 1224 participants completed the 21-day survey, and 273 completed the 90-day survey. The participants resided across Australia and were mostly women (4265/5062, 84.3%) with a mean age of 48.2 years (SD 14.1). The mean increase in intake was 0.48 servings, from 3.06 servings at baseline to 3.54 servings at the end of the 21-day challenge (t_1223_=8.71; *P*<.001). The variety of vegetables consumed also increased by 0.35 types per day (t_1123_=9.59; *P*<.001). No changes in intake and variety were found from day 21 to the 90-day follow-up. Participants with the highest app usage increased their vegetable intake by 0.63 (SD 2.02) servings per day compared with 0.32 (SD 1.69) servings per day for those with the lowest app usage. On the basis of multiple linear regression, gender; age; BMI; psychological variables of self-efficacy, attitudes, intentions, and action planning specific to vegetable intake; baseline vegetable intake; and active days of app usage accounted for 23.3% of the variance associated with the change in intake (*F*_9,1208_=42.09; *P*<.001). Baseline vegetable intake was the strongest predictor of change in intake (beta=−.495; *P*<.001), with lower baseline intake associated with a greater change in intake. Self-efficacy (beta=.116; *P*<.001), action planning (beta=.066; *P*=.02), BMI (beta=.070; *P*=.01), and app usage (beta=.081; *P*=.002) were all significant predictors of the change in intake.

**Conclusions:**

The VegEze app was able to increase intake by half a serving in a large sample of Australian adults. Testing the app in a real-world setting and embedding the consent process allowed for greater reach and an efficient, robust evaluation. Further work to improve engagement is warranted.

## Introduction

### Vegetables as a Target for Nutrition Intervention

Poor diet quality is a risk factor for the development of chronic disease [1] and, along with physical activity, a key modifiable behavioral target for health interventions. Poor quality diets are generally characterized by the inadequate consumption of fruits and vegetables, which are thought to account for 16.0 million (1.0%) disability-adjusted life years and 1.7 million (2.8%) deaths worldwide [2]. Hence, there have been many population-level initiatives addressing inadequate fruit and vegetable consumption that have achieved small to moderate improvements in intake [3-5]. Sustained improvements are more difficult to achieve, and in many countries such as Australia, the United States, and the United Kingdom, gaps between population intakes and national dietary recommendations persist [6-9].

Most large-scale population campaigns [7] and research interventions target fruit and vegetable consumption concurrently [10,11], with very few interventions focusing solely on vegetable intake. Targeting vegetables in isolation is needed as 95% of the Australian population do not meet the recommended intake levels for vegetables (compared with about 50% for fruits) [6]; there are more barriers for the consumption of vegetables than for fruits; there is a poorer understanding of serving sizes for vegetables [12], and when fruits and vegetables are targeted together, increases in consumption are largely associated with fruit and not vegetable intake [13].

### Benefits of Digital Technology for Behavior Change

Digital technologies are appealing for health-related behavior change interventions as they may overcome some of the limitations of traditional delivery approaches. For example, the ubiquitous nature of mobile phone ownership and its broad application and usage mean that mobile health (mHealth) interventions have the potential to reach large audiences at nearly any time or place. Mobile technology can also be highly interactive and can be used to deliver health-related information in a way that is engaging and rewarding. The ability to tailor content over time based on user inputs or objective measures (eg, wearable devices) can create personalization, which may increase engagement—the equivalent of exposure to or a dose of a traditional intervention—increasing the likelihood of success [14]. Mobile phones can also make self-monitoring and tracking easy, which, along with timely feedback [15,16], are key drivers and predictors of behavior change. From an implementation perspective, digital technologies may provide a cost-effective and scalable approach to deliver health interventions. This aspect is also appealing to researchers and practitioners as traditional public health interventions are often resource intensive, affecting the service delivery and potential impact.

### Mobile Health Interventions: Effectiveness and Validity

Preliminary evidence from mHealth interventions shows promise as they appear to be feasible and acceptable to users [17], but their success in changing behavior is less well established. To maximize the likelihood of success, digital interventions should be based on behavior change theories and utilize evidence-based content [18,19]. Many commercially available mobile phone apps are not evidence-based or grounded in behavior change theories [16,19], and those which do utilize the theories report larger intervention effects [16]. Even nutrition research apps that have been published and evaluated within the scientific domain rarely examine and adequately report the effects on dietary behavior change [20].

Research apps are typically evaluated using approaches such as randomized controlled trials, which emphasize internal validity as opposed to external validity. Such approaches provide robust evidence for efficacy in the study sample but have limited reach and provide little insight into their generalizability to the target population [21]. Apps that have been scientifically developed and tested also face challenges related to the slow pace of traditional research and lack of resources available for the eventual translation of these mHealth interventions for more widespread use [21,22].

### Objective

This study aimed to implement and evaluate the impact of a mobile phone app called VegEze, which aims to improve vegetable intake among Australian adults. The Australian Dietary Guidelines recommend “plenty of vegetables, including different types and colours” [23]; therefore, this app focused on increasing the amount and variety of vegetables consumed. Increasing the variety of vegetables has been shown to be an effective strategy to increase the consumption of vegetables in a single meal [24-26] but has not been targeted in previous large-scale digital interventions for adults. The target behavior for the VegEze app was *having 3 different types of vegetables at dinner.* A detailed description of the VegEze app and its development has been published [27].

To better capture the impact of this app in a real-world setting, the Reach, Effectiveness, Adoption, Implementation, and Maintenance (RE-AIM) framework was utilized. The framework has 5 components—reach, effectiveness, adoption, implementation, and maintenance—which are commonly used to translate research and understand impact and generalizability across populations [28,29]. This framework informed our key research questions, which are as follows:

- How many Australian adults are willing to participate in the VegEze intervention? (Reach)
- What is the impact of VegEze on increasing vegetable variety and intake? (Effectiveness)
- Who used the VegEze app most? (Adoption)
- How do participants use the features of the VegEze app and is app usage associated with success? (Implementation)
- Does the VegEze app support participants to maintain their consumption for a longer term? (Maintenance)

## Methods

### Study Design and Participant Recruitment

An uncontrolled, quantitative cohort study was used to assess the impact of the VegEze app on the daily intake and variety of vegetables, with an evaluation conducted after 21 and 90 days of the program. The VegEze research study launched in the Apple App Store (free of charge) on November 8, 2017. To facilitate timely dissemination of the results, data for inclusion in this evaluation were extracted after 6 months (app download and baseline survey were completed between November 2017 and May 2018).

There was associated media coverage, including free-to-air television and radio interviews as well as social media promotions from November 13, 2017. Emails were also sent to an existing database of people who had opted in to receive nutrition-related newsletters.

Participants were eligible for the study if they were aged 18 years and above, were living in Australia, owned a compatible iPhone Operating System (iOS )10 or 11 device (including iPhone or iPad) with an internet connection, and were willing and able to download the app and participate in the trial. Those who had any condition or self-prescribed diet that prevented them from consuming vegetables were excluded. This study was approved by the Commonwealth Scientific & Industrial Research Organisation (CSIRO) Health and Medical Human Research Ethics Committee Low Risk Review Panel (proposal number 13/2017) and was registered with the Australian New Zealand Clinical Trials Registry (ACTRN12618000481279).

### Data Collection

All data were collected via mobile phones. We leveraged the Apple ResearchKit framework with the app because of its innovative features in transforming the way a large-scale cohort study can be delivered via a mobile phone. The onboarding process, including participant information and electronic consent, and the surveys were embedded into the app. Evaluation surveys were administered at 3 time points: baseline, end of the 21-day challenge, and at the 90-day follow-up. Although the surveys were designed to be as short as possible, they were still able to capture critical outcomes and predictors of behavior. Standard demographic questions about age, gender, and self-reported height and weight were also asked in the baseline survey only.

### Outcomes

#### Primary Outcomes: Vegetable Consumption

The primary outcomes were vegetable intake (reported in servings per day) and vegetable variety (reported in types per day) and were assessed at each time point. Vegetable intake was assessed using a series of short questions from the previously validated CSIRO Healthy Diet Score survey [30,31], which asks about the usual frequency (daily, weekly, monthly, and never) and the amount consumed, in standard servings, within the timeframe selected. From this, servings per day was calculated and compared with the age- and gender-specific daily intake targets provided in the Australian Dietary Guidelines and was coded as meeting the guidelines if the intake was greater than or equal to the recommendation.

Vegetable variety was assessed in a single question asking about the number of different types of vegetables consumed in the past 2 days. Changes in vegetable intake and variety were calculated as end of challenge minus baseline consumption, where a positive value indicated an increase in consumption. One question asked about the frequency (always, usually, sometimes, and never) of achieving the target behavior, ie, having 3 different types of vegetables at dinner.

#### Psychological Variables

Attitudes, intentions, and self-efficacy are 3 well-established personal factors that have been shown to predict changes in health behavior [32,33]. More recently, it has been suggested that having action plans about how to implement the target behavior is also a critical predictor of behavior [32]. These 4 constructs were measured at each time point using previously validated scales, and participants responded on Likert scales. Nutrition self-efficacy was assessed using 5 questions [34]. Intentions [35] and attitudes [36] toward consuming more vegetables were assessed using 3 questions each, and action planning was assessed in 2 questions [37]. Reponses to questions were summed, and a higher score represented a higher amount of each construct.

#### App Usage

Firebase by Google was used to collect extensive app-related data, including screen views, engagement with notifications, and app events (ie, interactions such as tapping navigation buttons), which helped to explain the behavior of participants when using the app.

### Intervention: VegEze App Design

**Figure 1:**
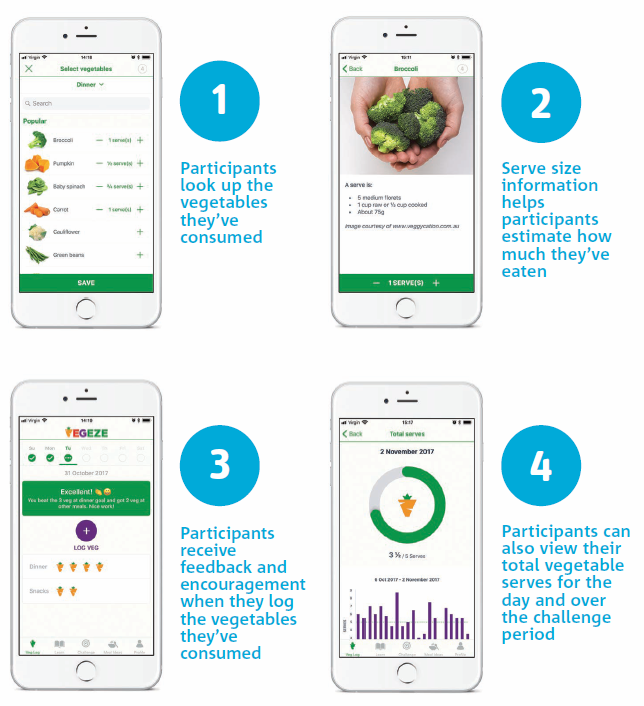
Vegetable log feature as a core component of the VegEze app.

A detailed description of the development process has been published previously [27]. VegEze is an Apple iOS–based app that was launched as a 21-day challenge to get Australians in the healthy habit of eating more vegetables, starting with 3 different types of vegetables in their evening or main meal. The app contained easy and fun ways to help establish this habit and encouraged individuals to monitor their vegetable intake with an easy-to-use tracker for logging the amount and types of vegetables consumed at each meal. Tracking intake was done through the vegetable log, which was the core feature of the app (Figure 1). Here, participants could search for vegetables they had consumed using an alphabetized list or a keyword search and record the amount they consumed in servings. Serving size information was available to assist with portion size estimation.

Two-way user feedback was also central to the app. On the home screen, users could review their daily progress for the current day (Figure 1). Feedback and motivational messages were also displayed here when participants logged their vegetable intake. The previous day’s log could be reviewed with a *check mark* indicating that the goal of 3 types was met. With a swipe from the home screen, users could also review their progress toward reaching the recommended number of servings per day (Figure 1).

**Figure 2:**
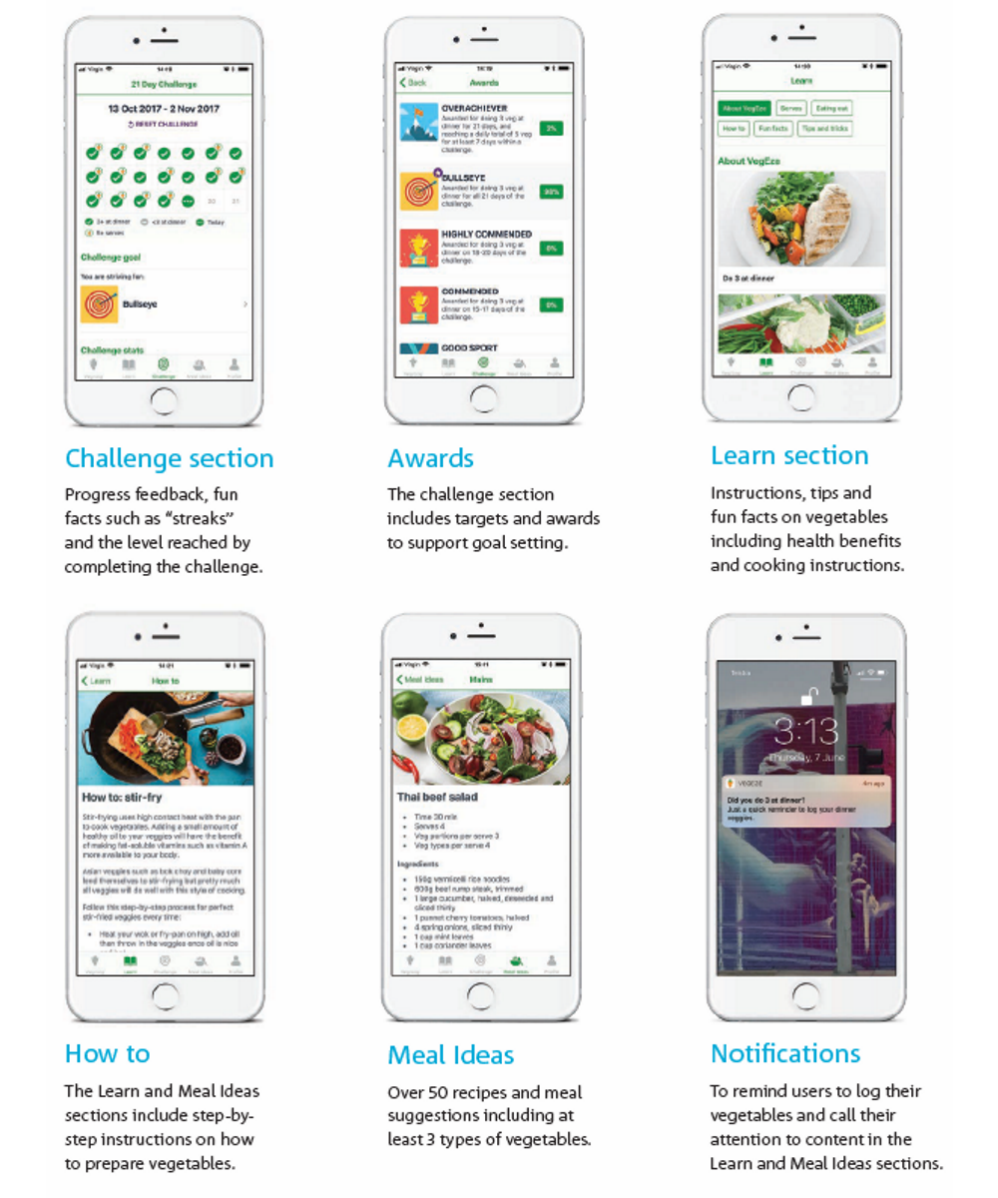
Other features of the VegEze app.

Other features of the app included a challenge and awards section that provided awards to support goal setting and further feedback on the level reached in the challenge. Content sections were divided into *Learn*, which provided tips and fun, evidence-based facts about vegetables; *How to*, which provided step-by-step instructions on how to prepare vegetables; and *Meal Ideas*, which provided over 50 recipes and meal suggestions for meeting the target behavior. Notifications to log intake and view content were also included at varying frequencies (Figure 2).

### Data Processing

The app usage and interaction logs were extracted from Big Query by Google into Excel files and processed in R. Any logs related to app updates, iOS updates, and errors were removed, and time stamps were converted from microseconds to the date and time format. Participants’ app log data for 21 days from the date of starting the challenge were used for the analysis.

Google Analytics was used to interpret high-level app usage for the entire cohort. The app usage data were linked via a unique ID to a custom database that recorded participation data such as vegetable logs, demographic information, and survey submissions. Structured Query Language queries were used to extract summarized participation data into Comma-Separated Values files for analysis.

Membership duration was calculated for individuals as the length of time (in days) between the start date of the challenge and the last date of app usage. The attrition rate of the app over 21 days of the program was calculated using the membership duration. The number of active days for which individuals visited the app (ie, app usage) and the feature usage (Home, Veg Lookup, Challenges, Notifications, Meal Ideas, and Learn) were calculated for users who had at least one log instance for that day.

Survey data were extracted from a service called SurveyGizmo into IBM SPSS Statistics files. Extreme outliers were removed based on vegetable intake that was greater than 12 servings of vegetables per day, reported at any time point (equivalent to the mean±3SD).

### Statistical Analysis

Descriptive statistics (means, standard deviations, and percentages) were used to describe the characteristics of the sample. We applied a per-protocol analysis to calculate the significance of the change in vegetable consumption between baseline and the end of the 21-day challenge. This change was tested using paired samples *t* tests for the sample as a whole, and within demographic subgroups of gender, age, and weight status. The differences in the change in consumption among demographic subgroups were assessed using 1-way analysis of variance. Multiple linear regression was used to assess the user characteristics (gender, age, BMI, baseline vegetable consumption, self-efficacy, attitudes, intentions, action planning, and app usage) that were associated with the change in consumption. User characteristics (predictors) were added to the model simultaneously. Descriptive statistics were used to describe app usage. Tertiles of app usage were created, and the mean changes in vegetable intake and variety were examined to understand how adaptation related to effectiveness. As an indication of maintenance of behavior change, the change in intake was examined between baseline and 90-day follow-up using paired samples *t* tests. Significance was assumed at a level of *P*<.05. Analyses were conducted using IBM SPSS Statistics version 25.

### Power

A previous review of digital interventions to increase vegetable intake, albeit in an adolescent population, reported increases of 0.1 to 0.4 servings of vegetables per day [10]. An a priori power calculation using the G*Power software [38] indicated that a sample of 272 people would be needed to detect a small effect size (*d*=0.2) with 95% power using a *t* test for the difference between 2 dependent means with alpha at .05. This was considered as the minimum number of participants needed per group to allow for a subgroup analysis. Variables such as age group and weight status have up to 4 subgroups; therefore, we aimed to retain 1100 participants until the end of the 21-day challenge. We expected 20% of the baseline sample to complete the 21-day survey; therefore, we aimed to recruit approximately 5000 participants. 

## Results

### Description of the Sample

**Figure 3:**
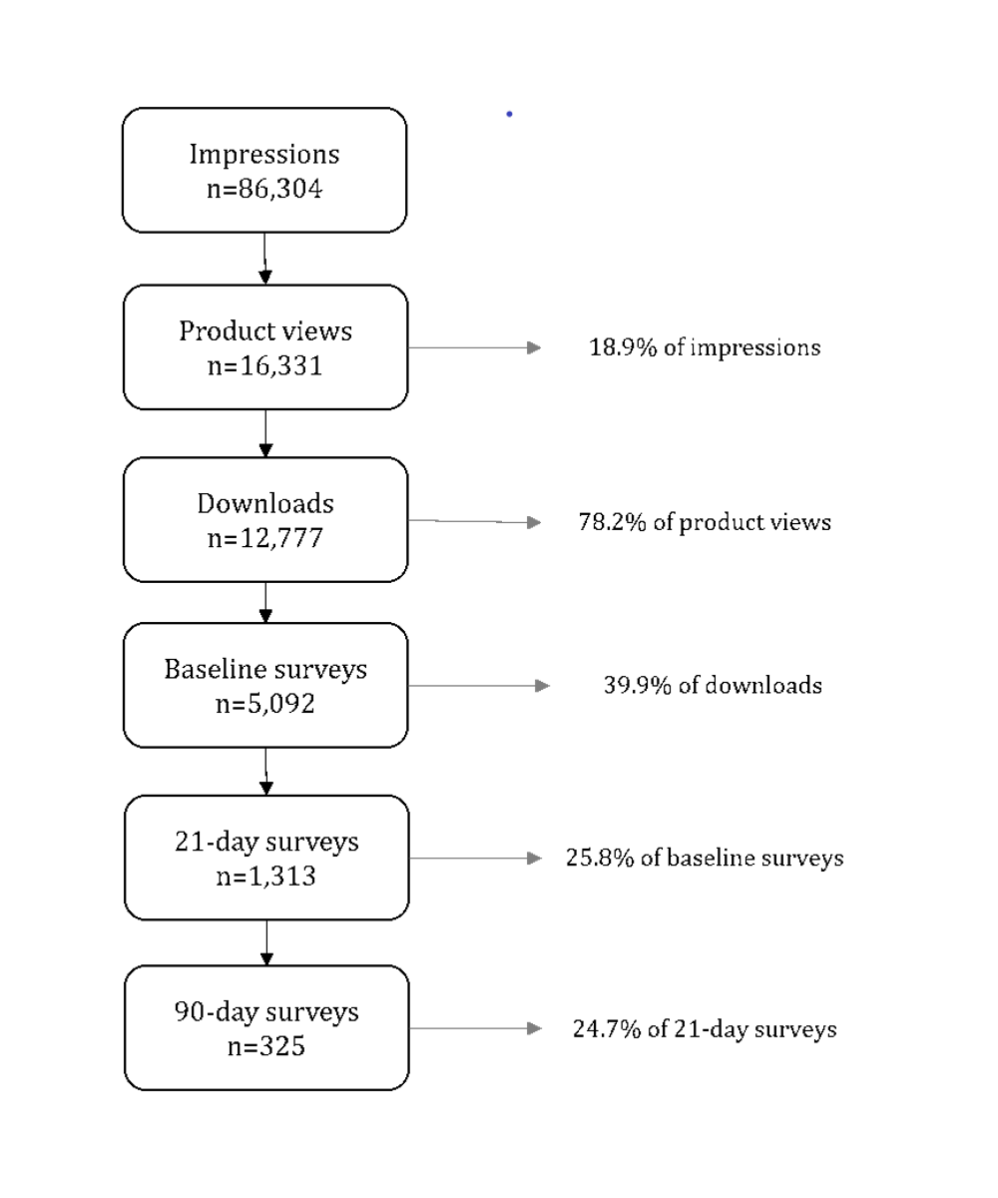
A flow chart of participants in the VegEze study.

The media coverage resulted in over 86,000 impressions within the App Store, over 16,000 product views, and 12,777 people downloading the VegEze app (Figure 3). Data were available from 5092 participants who completed the baseline survey, 1313 participants who had completed the 21-day end-of-challenge survey, and 325 participants who had completed the 90-day follow-up survey during this period. Given that the study was purely Web-based and there was no contact with participants outside of the app, reasons for nonparticipation at each stage were not collected.

Following data cleaning, survey data, including vegetable intake measures, were available for 5062 participants at baseline, 1224 participants at the end of the 21-day challenge, and 273 participants at the 90-day follow-up. At baseline, 4683 users had both app log and survey data, with 1219 having app and survey data at the end of the 21-day challenge.

Participants who installed the app and completed the baseline survey were from across Australia, were mostly women (4265/5062, 84.3%), and were aged between 31 and 70 years (4183/5062, 82.7%; the mean age of the sample was 48.2 years, SD 14.1). The sample had a greater proportion of obese adults than the Australian population (590/5062, 31.4% in the sample vs 27.5% in the Australian population) and consequently a lower proportion in the normal weight category (Table 1).

**Table 1:** Demographic characteristics of the baseline sample of participants and their comparison with the Australian population.

Demographic characteristics			Sample (N=5062), n (%)		Australian population^a^, %
**Gender**					
	Male	774 (15.29)		49.4	
	Female	4265 (84.25)		50.6	
	Unisex	23 (0.45)		—^b^	
**Age group (years)**					
	18-30	675 (13.33)		18.6	
	31-50	1997 (39.45)		37.7	
	51-70	2186 (43.18)		30.5	
	≥71	204 (4.03)		13.1	
**Weight status**					
	Underweight	52 (1.02)		1.7	
	Normal weight	1587 (31.35)		35.5	
	Overweight	1833 (36.21)		35.3	
	Obese	1590 (31.41)		27.5	
**Australian state or territory**					
	New South Wales	1529 (30.21)		32.2	
	Victoria	1484 (29.32)		24.9	
	Queensland	916 (18.10)		20.1	
	Western Australia	446 (8.81)		10.4	
	South Australia	435 (8.60)		7.4	
	Tasmania	76 (1.50)		2.3	
	Northern Territory	12 (0.24)		1.0	
	Australian Capital Territory	93 (1.84)		1.7	

^a^Australian population estimates were taken from the 2016 census, available from the Australian Bureau of Statistics [39].

^b^Data unavailable.

Average vegetable intake per day was 3.1 servings (SD 1.9) and 2.5 types (SD 1.3) at baseline. This translates to 14.4% (729/5062) meeting the Australian Dietary Guidelines recommendation for vegetable intake. Furthermore, 22.1% (1119/5062) of the sample reported of *always* having 3 different types of vegetable at dinner, and 64.5% (3265/5062) *always* or *usually* achieved this target.

### Impact on Vegetable Consumption

**Figure 4:**
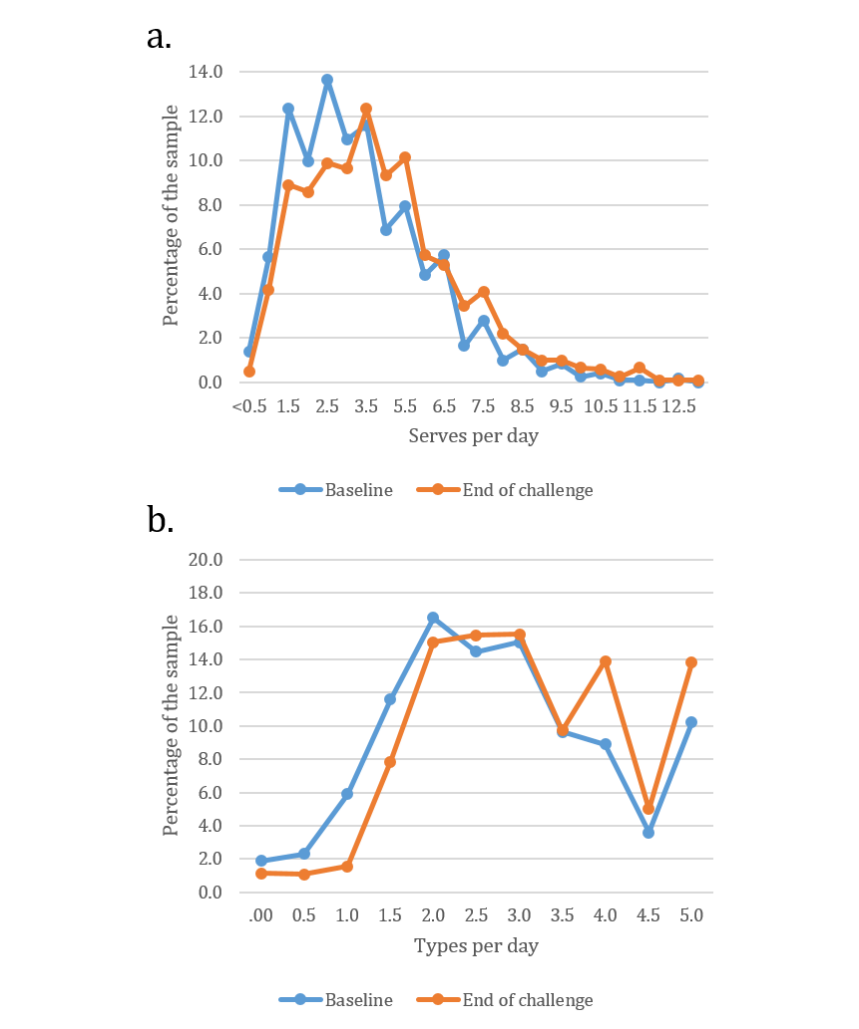
Distribution of (a) vegetable intake and (b) vegetable variety at baseline and at the end of the 21-day challenge.

In the sample that completed the 21-day survey (n=1224), the distribution of vegetable intake, in servings and types, at baseline and at the end of the 21-day challenge is shown in Figure 4. The mean increase in consumption was 0.48 servings, from 3.06 servings at baseline to 3.54 servings at the end of the challenge (t_1223_=8.71; *P*<.001). The variety of vegetables consumed also increased by 0.35 types per day (t_1123_=9.59; *P*<.001).

The changes in vegetable intake varied for different demographic groups. Women significantly increased their number of servings (0.51 servings; t_1077_=8.74; *P*<.001) and types (0.36 types; t_1077_=9.31; *P*<.001). The change in consumption for men was significant for the types (0.23 types; *P*=.02) but not the amount (0.25 servings; *P*=.13;). Significant increases in intake were also observed among participants in the age groups of 31 to 50 years and 51 to 70 years (0.50-0.53 servings; *P*<.001). All categories of weight status significantly increased intake (ranging from 0.40-0.53 servings; all *P*<.001; Table 2). The between-group differences for change in consumption were not significant for gender, age group, or weight status.

**Table 2:** Change in vegetable consumption (servings and types) at baseline and at the end of the 21-day challenge by demographic characteristics.

Demographic characteristics		Sample (N=1224), n (%)	Amount in servings				Number of varieties				
			Baseline consumption, mean (SD)	End of challenge (day 21), mean (SD)	Change, mean (SD)	*P* value		Baseline consumption, mean (SD)	End of challenge (day 21), mean (SD)	Change, mean (SD)	*P* value
**Gender^a^**											
	Male	139 (11.36)	3.06 (1.98)	3.30 (2.00)	0.25 (1.92)	.134		2.55 (1.26)	2.78 (1.13)	0.23 (1.17)	.02
	Female	1078 (88.07)	3.07 (1.72)	3.58 (1.92)	0.51 (1.92)	<.001		2.78 (1.24)	3.15 (1.19)	0.36 (1.29)	<.001
	Total	1224 (100.00)	3.06 (1.76)	3.54 (1.93)	0.48 (1.92)	<.001		2.75 (1.25)	3.10 (1.19)	0.35 (1.27)	<.001
**Age group (years)**											
	18-30	95 (7.76)	3.08 (1.68)	3.30 (1.87)	0.22 (1.74)	.225		2.73 (1.33)	3.10 (1.17)	0.38 (1.30)	.01
	31-50	411 (33.58)	2.75 (1.63)	3.28 (1.76)	0.53 (1.83)	<.001		2.75 (1.26)	3.09 (1.20)	0.34 (1.32)	<.001
	51-70	654 (53.43)	3.24 (1.81)	3.74 (2.01)	0.50 (2.01)	<.001		2.77 (1.22)	3.12 (1.19)	0.35 (1.26)	<.001
	≥71	63 (5.15)	3.23 (1.88)	3.54 (2.00)	0.31 (1.79)	.172		2.57 (1.32)	2.94 (1.08)	0.37 (1.03)	.01
**Weight status^a^**											
	Normal weight	392 (32.03)	3.01 (1.68)	3.40 (1.89)	0.40 (1.84)	<.001		2.90 (1.22)	3.27 (1.17)	0.37 (1.19)	<.001
	Overweight	436 (35.62)	3.08 (1.78)	3.61 (1.98)	0.53 (1.99)	<.001		2.69 (1.26)	3.05 (1.19)	0.36 (1.29)	<.001
	Obese	386 (31.53)	3.10 (1.80)	3.61 (1.91)	0.51 (1.94)	<.001		2.67 (1.24)	2.99 (1.19)	0.32 (1.33)	<.001

^a^An inadequate sample size to report on unisex (n=6) or underweight categories (n=9).

In this sample, the proportion of the sample meeting the dietary guidelines recommendation for vegetable consumption increased from 15.9% (195/1224) at baseline to 22.6% (277/1224) at the end of 21 days. The proportion of the sample reporting of *always* having 3 different types of vegetables at dinner increased from 23.2% (284/1224) at baseline to 28.8% (353/1224) at the end of 21 days, and the percentage who were *always* or *usually* achieving this target behavior increased from 68.2% (835/1224) to 81.3% (995/1224).

### Impact on Psychological Factors

There was a small but significant increase in positive attitudes toward eating a greater variety of vegetables during the challenge period, albeit there was a highly positive attitude at baseline (3.84 to 3.87 points out of 4; t_1223_=2.85; *P*=.005). Nutrition self-efficacy also increased significantly (14.54 to 15.04 out of 20; t_1223_=6.09; *P*<.001), as did planning related to eating vegetables (3.27 to 3.60 points out of 6; t_1223_=7.01; *P*<.001). The intention to eat a greater variety of vegetables (4.91 to 4.60 points out of 6; t_1223_=−9.48; *P*<.001) and intention to use the app (5.33 to 4.49 points out of 6; t_1223_=−19.20; *P*<.001) decreased significantly during the 21-day challenge.

### App Usage Statistics

**Figure 5:**
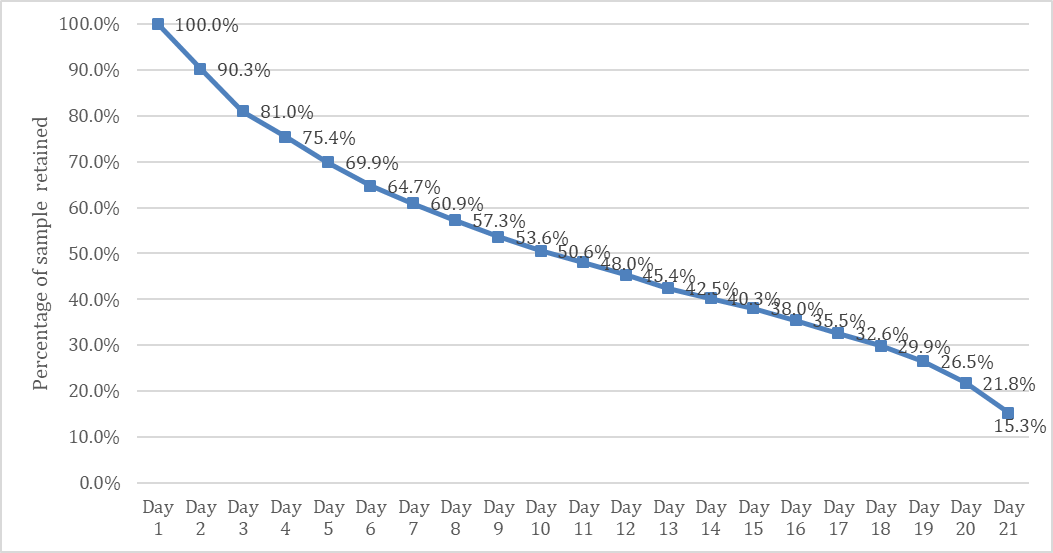
Participant attrition over the 21-day challenge (n=4683).

The attrition curve for the usage of the app is shown in Figure 5. There was a gradual reduction in the percentage of the sample retained. By day 10 of the challenge, about half the sample was using the app, and 21 days after completing the survey, 15.3% (719/4683) of participants were still using the app.

On average, participants actively used the app for 6.3 days out of the 21 days of the challenge. Furthermore, 49.2% (2304/4683) actively used the app for 2 to 7 days, 19.1% (894/4683) used the app for 8 to 14 days, 11.6% (543/4683) for 15 to 20 days, and 1.2% (56/4683) used the app every day during the 21-day challenge period (Table 3). Women were more likely than men to use the app for more than 7 days during the challenge (1302/3956, 32.9% vs 184/707, 26.0%; data not shown). On average, women actively used the app for 6.4 days during the challenge compared with 5.6 days for men (*P*=.001). Those in the oldest 2 age groups used the app more than those in the 2 younger age groups (5.1 days vs 7.1 days for age groups 19-50 years and ≥51 years, respectively; *P*<.001). App usage did not differ among categories of weight status.

**Table 3:** Percentage of the sample actively using the VegEze app, for the baseline sample and for those that completed the program.

App usage		Baseline (n=4683)	Completers (n=1219)
**Frequency of usage (days), %**			
	1	19.0	2.1
	2-7	49.2	18.4
	8-14	19.1	38.6
	15-20	11.6	36.8
	21	1.2	4.1
**Feature usage, days**			
	Total usage	6.3	12.5
	Home screen	6.1	12.3
	Veg Lookup	3.7	8.3
	Challenges	1.3	2.4
	Meal Ideas	0.9	1.6
	Notifications	1.0	2.0
	Learn	0.6	1.1

The average active usage was higher among the participants who had app data and completed the 21-day survey (n=1219/1224), ie, 12.5 days out of the 21-day challenge. Furthermore, 36.8% (449/1219) of this sample used the app for 15 to 20 days, and 4.1% (50/1219) used the app every day during the 21-day challenge (Table 3).

**Figure 6:**
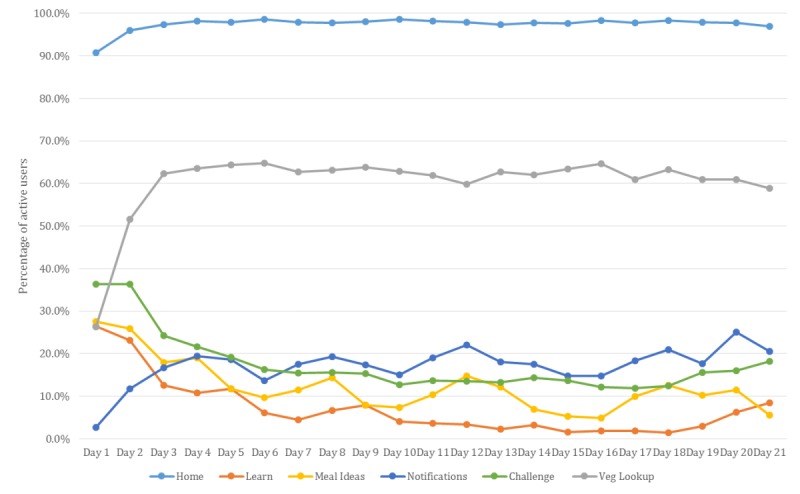
Percentage of active users using app features throughout the 21-day challenge period (n=4683).

Figure 6 shows the percentage of active users (n=4683) for each feature. Aside from the home screen, the most commonly used feature of the app was the Veg Lookup section. The usage of this feature increased from 26.3% (1232/4683) on day 1 to 62.3% (2918/4683) on day 3 and then stayed relatively stable, with about 60% to 63% of active users using this feature on any day of the challenge. Viewing the notifications appeared to increase in the second half of the challenge period from about 15% of active users in the first half of the challenge period to about 20% in the later part. In contrast, the percentage of users viewing the challenge screen dipped from 36% of users on days 1 and 2 to between 12% and 15% at roughly day 10 and then peaked slightly again in the last few days of the challenge. The Meal Ideas and Learn sections of the app were used least frequently, with usage ranging from 5% to 25% of active users on any particular day during the challenge (Figure 6).

### Associations Between App Usage and Vegetable Consumption

**Figure 7:**
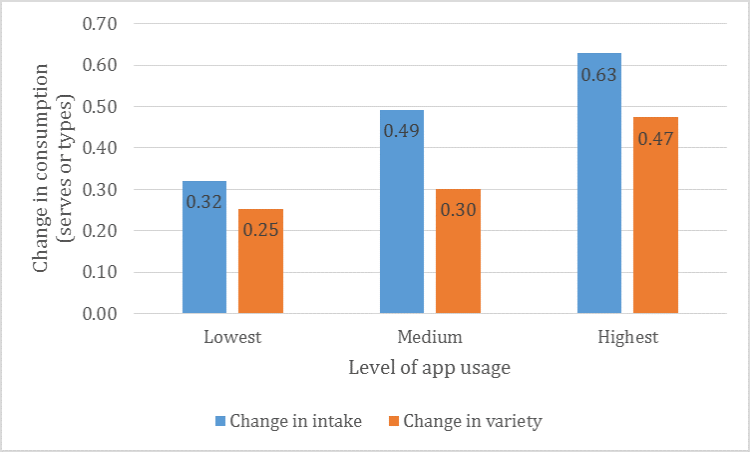
Change in vegetable consumption by level of app usage* (n=1219). *Lowest tertile of app usage: active days ranged from 1 to -10 days, average 6.4 days; medium app usage: active days ranged from 11 to -15 days, average 13.1 days; and high app usage: active days ranged from 16 to -21 days, average 18.3 days.

When app usage was divided into tertile groups, it was found that participants with the highest usage had actively used the app almost every day during the 21-day challenge. This group increased their vegetable intake by 0.63 servings (SD 2.02) per day over the 21-day challenge compared with 0.32 servings (SD 1.69) per day for those with the lowest app usage (difference=0.31 servings; *P*=.06, Figure 7). Changes in the variety of vegetables consumed also increased in a stepwise manner with increasing app usage. Participants who used the app the most increased their consumption variety by 0.47 types (SD 1.26) per day, which was significantly more than those who used the app the least and increased their intake by 0.25 (SD 1.28) types per day (difference=0.22 types; *P*=.03, Figure 7).

On the basis of multiple linear regression, gender; age; BMI; psychological variables of self-efficacy, attitudes, intentions, and action planning specific to vegetables; baseline vegetable intake; and active days of app usage accounted for 23.3% of the variance associated with the change in intake (*F*_9,1208_=42.09; *P*<.001). Baseline vegetable intake was the strongest predictor of change in intake (beta=−.495; *P*<.001), with lower baseline intake associated with greater change in intake. Self-efficacy (beta=.116; *P*<.001), action planning (beta=.066; *P*=.02), BMI (beta=.070; *P*=.01), and app usage (beta=.08; *P*=.002) were all significant predictors of change in intake. The same model was used to predict a change in variety. This model explained 32.3% of the variance (*F*_9,1208_=65.46; *P*<.001). Baseline vegetable variety (beta=−.582; *P*<.001) and app usage (beta=.110; *P*<.001) were the strongest predictors of change in variety. Baseline attitudes toward vegetables (beta=.058; *P*=.02) and gender (beta=.050; *P*=.04) were the other significant predictors.

### Maintaining Behavior Change for a Longer Term

Of those who completed the 90-day survey (n=273), 93% (254/273) were women, 25.6% (70/273) were aged between 31 and 50 years, 65.2% (178/273) were aged between 51 and 70 years, 34.1% (93/273) were overweight, and 36.6% (100/273) were obese. In this sample, vegetable intake increased significantly from 3.1 servings at baseline to 3.8 servings (average increase 0.68 servings; *P*<.001). Interestingly, this increase occurred during the first 21 days and was maintained during the follow-up period with an increase of 0.53 servings from baseline to the end of the 21-day challenge (*P*<.001) and a nonsignificant increase of 0.15 servings between days 21 and 90. Variety also increased significantly between baseline and the end of 21 days (average increase 0.48 types; *P*<.001) but not further during the extended follow-up period.

In this sample, the percentage of participants meeting the Australian Dietary Guidelines for vegetables increased from 16% (44/273) at baseline to 25% (68/273) and 26% (72/273) at the end of 21 and 90 days, respectively. Participants reporting of *always* having 3 different types of vegetables at dinner increased from 22.3% (61/273) at baseline to 32.2% (88/273) at the end of the 21-day challenge and then to 36.3% (99/273) at the end of the 90-day follow-up period. The percentage who *always* or *usually* had 3 different types of vegetables increased from 71.8% (196/273) at baseline to 83.5% (228/273) and 87.5% (239/273) at the end of the 21-day challenge and 90-day follow-up, respectively.

## Discussion

### Principal Findings

The VegEze app was designed to be an engaging 21-day challenge to increase the amount and variety of vegetables consumed by Australian adults. Central to this was a clear and specific behavioral target of having 3 different types of vegetables at dinner each day. At baseline, 22% of the sample reported *always* doing this and 68% reported *always or usually* doing this, which increased to 29% and 81%, respectively, at the end of the challenge. The dissemination pathway for this study was chosen to maximize its reach, and subsequently, the evaluation framework aimed to understand the impact and generalizability of the results within the population more broadly. The results of this study are discussed using the RE-AIM framework [28] and within the context of other scientific literature.

#### Reach—How Many People Were Willing to Participate?

This research app was made available through the Apple App Store, and the use of ResearchKit negated the need for any face-to-face contact and streamlined the consent process. This novel approach combined with a structured promotional campaign allowed us to reach a large national sample of over 5000 participants within a relatively short period. That said, only 40% of those who downloaded the app completed the baseline survey. Therefore, including the evaluation surveys as a compulsory part of the onboarding process appeared to be a barrier to the overall uptake. This may be because of the time burden and delayed gratification associated with completing the surveys for people who simply wanted to download and explore the app.

Compared with the Australian population, the sample of people reached by the recruitment process was largely women (84% in the sample vs 51% in the Australian population) and slightly younger (4% aged over 70 years in the sample vs 13% of the Australian population). Other characteristics were fairly similar to the broader population. Furthermore, the 1200 participants who completed surveys at the beginning and at the end of the challenge may represent a biased sample of those more motivated to participate in research, and the results may overstate the likely impact on intake. Therefore, whether the reported changes in consumption are generalizable to the population more broadly is unknown.

#### Effectiveness—What Was the Impact on Vegetable Intake?

In terms of the impact of the intervention on critical outcomes, the app showed great promise. Evaluation data indicated that the 1224 users who completed the survey at the end of the challenge reported an average increase of 0.5 servings in daily vegetable intake and an increase in variety of 0.35 types over 21 days. This change is consistent with, or greater than, the changes reported by large-scale population campaigns [4] and other digital interventions focused on increasing vegetable intake [10,40]. A recent systematic review of electronic health and mHealth interventions for young adults reported increases in vegetable intake of between 0.1 and 0.4 servings per day [10]. Another study not included in the aforementioned review, developed and tested an app to increase vegetable intake among overweight adults who were already participating in a weight loss study. Participants with access to the app (n=68) increased their vegetable consumption by 0.8 servings over 35 days (5 weeks) compared with a control group who reported a decrease in consumption [15]. It is possible that people participating in weight management programs may be more motivated to change their eating behaviors as they are gaining the added and immediate reinforcement of weight loss.

We found positive changes in attitudes, self-efficacy, and action planning during the challenge period, which can help change behavioral intention into action [41]. However, it should be noted that attitudes toward eating a greater variety of vegetables were high at baseline, a likely indication of a motivated sample. We also found that higher levels of self-efficacy and action planning at baseline were associated with greater increase in vegetable consumption. Self-efficacy has been directly related to health behavior [33]. Its effect can also be indirect through its impact on goals. Individuals with higher self-efficacy, ie, who are more confident in their ability, may challenge themselves more by setting higher goals and focus on opportunities rather than obstacles in carrying out the specific behavior [42]. Literature also suggests that those with higher nutrition-related self-efficacy are less likely to relapse to their previous unhealthy habits [43]. Therefore, changes in all these variables are considered promising for future vegetable consumption. It is interesting to note that we also found a decrease in individuals’ reported intention to eat a greater variety of vegetables. These findings are consistent with other evaluations of app-delivered programs [44]. There are several possible reasons for this. For example, the VegEze app centered around a short challenge, and at the end, participants may have felt as though they had completed the program and no longer needed to keep improving and/or keep using the app and therefore reported a lower intention. They may have also felt successful in their behavior change endeavors and therefore no longer required as much motivation to continue performing the target behavior. Finally, motivation is critical for initiating new behaviors, but as the behavior becomes routine, different factors may be needed to continue to support performing the behavior.

#### Adoption—Who Was Most Likely to Use the App?

It was a purposeful decision to test this app in the real world; therefore, participants of this study used the app in representative settings. With regard to the representativeness of those who engaged with the program, women and those aged 51 years and above used the app more than men and younger adults. Despite recruiting participants through multiple channels—such as free-to-air television and radio coverage, social media, and an existing database—similar to the findings of previous health-related interventions, the majority of this study sample was women. Traditionally, mobile phone usage has been higher in younger adults, but more recently, the largest growth has been among older Australians [45]. Consistent with this, we found that, on average, the older participants used VegEze for an extra 2 days in the 21-day challenge period. The benefits of consuming more vegetables may also be perceived as more beneficial for older participants as they are seeking to improve their health. Engagement with technology is increasing across all age groups, and the appeal of apps as a delivery platform technology should be considered in health intervention regardless of the age profile of targeted users.

#### Implementation—App Usage Patterns and Association With Success

Baseline vegetable consumption and app usage were the 2 strongest predictors of increased vegetable intake and variety. Participants with the highest usage, ie, using the app almost every day (16-21 days; average 18 days), increased their consumption by twice as much as those with the lowest app usage. The cause and effect cannot be assumed; however, it is encouraging that higher engagement with the app was associated with a more positive outcome in the behavior of interest. The fact that lower vegetable consumers reported greater increases in consumption was also promising. Although all Australians need to increase their vegetable intake to meet recommendations [6], arguably those with the lowest intakes have the greatest need for support and will benefit the most from intervention. It is not always the case that interventions work in those that need it most. For example, Mummah et al [15] reported that baseline consumption was a significant moderator of intervention effect whereby the impact of their app on vegetable intake was the greatest among those who reported higher intakes at baseline.

#### Maintenance—Is Behavior Change Sustained?

Long-term maintenance of behavior is important for realizing the health benefits associated with higher vegetable intake. Initial increases in vegetable intake were sustained up till a 3-month follow-up for which 87% of the sample reported of *always* or *usually* including 3 different types of vegetables at dinner, and one-quarter of the sample was meeting the dietary guidelines recommendations. Nationally, it is estimated that less than 5% of Australian adults have adequate vegetable intake [6], and public health and policy initiatives are seeking novel approaches to shift this persistent population trend. To determine whether this app has been truly effective in long-term behavior change, we would need to extend the follow-up to 6 months or more.

Other digital interventions have not been able to sustain changes in vegetable intake over 3 months [40]. We found that the increases in intake were maintained despite a reduction in engagement with the app. It is possible that users may reduce their reliance on the app once their goal has been achieved and a new way of eating has been formed. This is possible given the purposeful simplicity of the target behavior. Participants started the challenge with a high level of intention to increase their vegetable intake, and it is possible that as they progressed; they felt they were successful in their behavior change endeavors and therefore no longer required as much motivation from the app features, which may be why the intention to use the app fell. As a behavior becomes routine, different factors may be needed to continue maintenance [32]. Similarly, apps may need to evolve with the users’ stage of change.

### Limitations

There are limitations to this study that warrant discussion. The study design was chosen to maximize reach and replicate a real-world setting, but a cohort study evaluation is not as strong to show efficacy as other methods such as a randomized controlled trial, and therefore caution is required in the interpretation of results. The subsample of participants who completed the follow-up survey was less than half of those doing the baseline survey, and data imputation was not used to account for missing data. Therefore, the change in vegetable consumption reported here is likely to overstate the potential of this app on intake more generally. Australia is largely a mobile phone market dominated by Apple and Samsung, with 42% and 35% of the market share, respectively [45]. For pragmatic and financial reasons, we decided to initially test this app on the iOS only, which limited the potential sample. Despite making the app as accessible as possible through the App Store, we still recruited a largely female sample, and those who completed the evaluation surveys are likely to represent the more motivated, health-interested users. It remains a challenge to engage with other subgroups of the population who are also likely to benefit from technology-based nutrition interventions.

Maintaining interest in an app is difficult. Only 15% of the sample actively used the app for the entire 21 days of the challenge. Nonetheless, the attrition rate observed here was similar to what has been reported elsewhere [46]. Furthermore, to facilitate timely dissemination of results, the recruitment window and study time frames for this study were short. We do not know the optimal exposure time needed for an intervention message to achieve the greatest result, and in fact, this may differ depending on the behavior of interest. Although the follow-up data suggest that behavior change can be maintained, we would require additional time to demonstrate a more sustained impact on vegetable intake in a larger, more diverse sample.

### Conclusions

Inadequate vegetable intake is a global problem, and the health impact of increased vegetable intake is well known, meaning, there is a real need for novel strategies and interventions that achieve successful increase in vegetable consumption. The VegEze app was designed as a 21-day challenge to increase vegetable consumption, and results indicate that focusing on a specific behavioral target around increasing variety was successful in increasing the amount of vegetables consumed. We were able to shift the distribution of intake in a large sample and increase the average consumption by half a serving. Utilizing Apple’s ResearchKit allowed us to embed the evaluation process in the app and still place it in the App Store, contributing to the overall reach, generalizability, and rigorous evaluation of outcomes. Future research apps may also choose this approach to allow for more frequent evaluation of mHealth nutrition interventions in an uncontrolled real-world setting. Given that app usage was associated with successful behavior change, further work to improve engagement is warranted.

## Abbreviations

CSIRO: Commonwealth Scientific & Industrial Research Organisation
iOS: iPhone Operating System
HIAL: Horticulture Innovation Australia Ltd
mHealth: mobile health
RE-AIM: Reach, Effectiveness, Adoption, Implementation, and Maintenance
